# Re-irradiation combined with bevacizumab for recurrent glioblastoma beyond bevacizumab failure: survival outcomes and prognostic factors

**DOI:** 10.1038/s41598-023-36290-2

**Published:** 2023-06-09

**Authors:** Weir-Chiang You, Hsu-Dung Lee, Hung-Chuan Pan, Hung-Chieh Chen

**Affiliations:** 1 Department of Radiation Oncology, Taichung Veterans General Hospital, 1650, Tawain Blvd Section 4, Taichung, 40704 Taiwan; 2 Department of Neurosurgery, Taichung Veterans General Hospital, Taichung, Taiwan; 3 Department of Radiology, Taichung Veterans General Hospital, Taichung, Taiwan

**Keywords:** CNS cancer, CNS cancer

## Abstract

The combination of re-irradiation and bevacizumab has emerged as a potential therapeutic strategy for patients experiencing their first glioblastoma multiforme (GBM) recurrence. This study aims to assess the effectiveness of the re-irradiation and bevacizumab combination in treating second-progression GBM patients who are resistant to bevacizumab monotherapy. This retrospective study enrolled 64 patients who developed a second progression after single-agent bevacizumab therapy. The patients were divided into two groups: 35 underwent best supportive care (none-ReRT group), and 29 received bevacizumab and re-irradiation (ReRT group). The study measured the overall survival time after bevacizumab failure (OST-BF) and re-irradiation (OST-RT). Statistical tests were used to compare categorical variables, evaluate the difference in recurrence patterns between the two groups, and identify optimal cutoff points for re-irradiation volume. The results of the Kaplan–Meier survival analysis indicated that the re-irradiation (ReRT) group experienced a significantly higher survival rate and longer median survival time than the non-ReRT group. The median OST-BF and OST-RT were 14.5 months and 8.8 months, respectively, for the ReRT group, while the OST-BF for the none-ReRT group was 3.9 months (*p* < 0.001). The multivariable analysis identified the re-irradiation target volume as a significant factor for OST-RT. Moreover, the re-irradiation target volume exhibited excellent discriminatory ability in the area under the curve (AUC) analysis, with an optimal cutoff point of greater than 27.58 ml. These findings suggest that incorporating re-irradiation with bevacizumab therapy may be a promising treatment strategy for patients with recurrent GBM resistant to bevacizumab monotherapy. The re-irradiation target volume may serve as a valuable selection factor in determining which patients with recurrent GBM are likely to benefit from the combined re-irradiation and bevacizumab treatment modality.

## Introduction

Glioblastoma multiforme (GBM) is a highly aggressive primary brain tumor with a 5-year relative survival rate of only 6.9%^1^. Although bevacizumab, a monoclonal antibody targeting vascular endothelial growth factor (VEGF), has received FDA approval for treating recurrent GBM, its effectiveness in progressive GBM after initial therapy remains limited^2–5^. While bevacizumab is a valuable second-line treatment for patients with GBM who have failed other therapies, such as radiotherapy, temozolomide, and lomustin^6^, there is still a pressing need for innovative therapeutic approaches for recurrent GBM following bevacizumab failure.

Re-irradiation in combination with bevacizumab has emerged as a promising treatment option for recurrent GBM. This is partly due to advances in radiation therapy techniques, which allow for better sparing of critical structures and reduced risk of neurologic toxicities^7^. Although potential late neurologic toxicities may be associated with re-irradiation, careful patient selection and treatment planning can help minimize these risks^8,9^. Notably, the recent phase II NRG Oncology/RTOG 1205 trial demonstrated a significant survival benefit in recurrent GBM when treated with re-irradiation and bevacizumab, accompanied by acceptable toxicity profiles^10^. Additionally, bevacizumab has been found to reduce the incidence of radionecrosis in high-grade glioma patients treated with re-irradiation^11^. These findings suggest that re-irradiation with bevacizumab may be a viable treatment option for patients with recurrent GBM after bevacizumab failure, addressing an unmet need in the field.

Despite the scarcity of treatment options for recurrent GBM following bevacizumab failure, the optimal approach relies on various factors^12–15^. Prior research has shown no survival benefit in continuing bevacizumab therapy for recurrent GBM beyond disease progression^16,17^. However, comprehensive studies comparing the introduction of re-irradiation during bevacizumab failure to the best supportive care are lacking. Consequently, this study seeks to evaluate the effectiveness of re-irradiation combined with bevacizumab in patients with second progression GBM resistant to bevacizumab monotherapy, and to explore factors that may affect survival outcomes. By examining the impact of re-irradiation, this research aims to provide valuable insights into optimizing treatment strategies for this challenging patient group.

## Materials and methods

### Data source and patient selection

This retrospective study included 78 consecutive patients with recurrent GBM who underwent surgery and postoperative chemoradiotherapy at a hospital between December 2009 and December 2019. Following the first recurrence, patients received bi-weekly 100 mg/m^2^ bevacizumab monotherapy. Progressive disease was confirmed through MRI scans, as defined by Macdonald and RANO criteria. Patients' MRIs were followed at three-month intervals until death. Regular MRI scans confirmed the second progression. Patients who did not receive bevacizumab therapy or treatments other than bevacizumab at the first progression were excluded (as shown in Supplementary 1). Of the remaining patients, those who developed a second progression after single-agent bevacizumab therapy were divided into two groups: 35 underwent best supportive care (non-ReRT group), and 29 received bevacizumab and re-irradiation (ReRT group). The selection of re-irradiation was based on patients' performance and preferences, as well as factors such as recurrence volume and location and the risk of radionecrosis. The Institutional Review Board (IRB) of Taichung Veterans General Hospital approved this retrospective study. Informed consent was obtained from all subjects, and all experiments were conducted in compliance with the relevant guidelines and regulations established by the IRB.

### Data collection

A predefined protocol was used to collect patient characteristics and tumor/treatment-related parameters from medical records. The response to bevacizumab treatment was evaluated by comparing MRI scans before and after the initial treatment to determine the Objective Response Rate (ORR). Karnofsky Performance Scale (KPS) scores were obtained at the time of the second progression, indicating bevacizumab failure. The failure pattern of bevacizumab was classified as locoregional, leptomeningeal spread (LMS), or both. LMS was diagnosed based on the appearance on MRI as linear or nodular lesions with high signal intensity on T2-weighted images and low signal intensity on T1-weighted images that were enhanced with gadolinium contrast agent^18^. Other treatment-related parameters, such as patterns of bevacizumab failure, characteristics of targets for re-irradiation, the extent of resection (EOR), and re-irradiation target volume, were also collected. To evaluate the coverage of tumor sites and normal brain tissues, a Dose Volume Histogram (DVH) was plotted. The normal brain was defined by total brain volume, excluding the planning target volume (PTV) or gross tumor volume (GTV). Brain V50, V60, and V80, which refer to the percentage of the normal brain receiving at least 50, 60, and 80 Gy, respectively, were calculated based on the whole brain volume. 80 Gy, respectively, were calculated based on the whole brain volume.

### Endpoints and statistical analyses

The study's primary endpoint was to investigate the survival benefit of adding re-irradiation to continuing bevacizumab in patients with recurrent GBM after bevacizumab failure. The secondary endpoint was to explore factors that may help clinicians choose treatment options. The study measured overall survival time after bevacizumab failure (OST-BF) and re-irradiation (OST-RT), censoring surviving patients on dates without follow-up. Statistical tests such as Fisher's exact test, independent *t*-test, Chi-square test, and Mann–Whitney U test were used to compare categorical variables on patient characteristics and evaluate the difference in recurrence patterns between the two groups. ROC analysis was used to identify the optimal cutoff points for re-irradiation volume to determine which patients may have better survival. Survival curves were estimated using the Kaplan–Meier method and log-rank tests to determine their significance. Univariate and multivariate analyses were performed using Cox proportional hazards models. All statistical tests were conducted using SPSS version 19 software, with *p*-values less than 0.05 considered statistically significant.

## Results

The study assessed differences in patient characteristics and treatment-related parameters between the two groups, as shown in Table 1. No significant differences were observed in gender, age, tumor location, multifocal GBM, the extent of resection during the first surgery, IDH1-R132H mutation (available for 25 patients), V50, or V60 between the two groups. However, the ReRT group had a significantly higher proportion of patients with neurological symptoms at bevacizumab failure (82.8% vs. 60.0%, *p* = 0.047) and a higher proportion of patients who underwent re-surgery after bevacizumab failure (37.9% vs. 14.3%, *p* = 0.030). Moreover, the ReRT group demonstrated a significantly higher ORR to bevacizumab than the non-ReRT group (complete response, 67.9% vs. 14.3%, *p* = 0.004). Additionally, a significantly higher proportion of LMS before or after bevacizumab therapy was observed in the ReRT group compared to the non-ReRT group (69.0% vs. 40.0%, *p* = 0.021). The study found no significant differences in other variables between the two groups.

**Table 1:** Demographic data and bevacizumab response in two groups.

Items	No ReRT (n = 35)	ReRT (n = 29)	*p* value
Gender			0.609
Male	22 (62.9%)	20 (69.0%)	
Female	13 (37.1%)	9 (31.0%)	
Age, years	55.29 ± 10.00	50.55 ± 13.42	0.122
RL			0.393
Right	18 (51.4%)	18 (62.1%)	
Left	17 (48.6%)	11 (37.9%)	
Site			0.972
Frontal	7 (20.0%)	5 (17.2%)	
Parietal	8 (22.9%)	6 (20.7%)	
Occipital	4 (11.4%)	3 (10.3%)	
Temporal	13 (37.1%)	11 (37.9%)	
Others	3 (8.6%)	4 (13.8%)	
Multifocal GBM			0.741
None	28 (80.0%)	25 (86.2%)	
Yes	7 (20.0%)	4 (13.8%)	
1st Surgery EOR			0.174
GTR	17 (48.6%)	19 (65.5%)	
STR-PR	18 (51.4%)	10 (34.5%)	
IDH1-R132H mutation			1.000
None	10	14	
Yes	0	1	
Re-irradiation dose/fractions			
3500 cGy/10 fractions		1 (3.4%)	
3600 cGy/20 fractions		1 (3.4%)	
4200 cGy/6 fractions		1 (3.4%)	
4600 cGy/20 fractions		26 (89.7%)	
V50 (%)	28.0% ± 10.4%	25.6% ± 12.7%	0.403
V60 (%)	21.4% ± 8.6%	19.6% ± 10.4%	0.474
V80 (%)	0%	9.4% ± 5.7%	
Re-surgery			
None	30 (85.7%)	18 (62.1%)	
First recurrence	0 (0.0%)	0 (0.0%)	
2nd Recurrence	5 (14.3%)	11 (37.9%)	
KPS at bevacizumab failure			0.248
60	6 (17.1%)	1 (3.4%)	
70	20 (57.2%)	19 (65.6%)	
80	9 (25.7%)	8 (27.6%)	
90	0 (0.0%)	1 (3.4%)	
Neurologic symptoms at bevacizumab failure	21 (60.0%)	24 (82.8%	0.047*
Re-surgery after bevacizumab failure	5 (14.3%)	11 (37.9%)	0.030*
Bevacizumab ORR			0.006**
Complete response	8 (14.3%)	19 (65.5%)	
Partial response	11 (31.4%)	4 (13.8%)	
Stable	1 (2.9%)	1 (3.4%)	
Progression	15 (42.9%)	5 (17.2%)	
Bevacizumab failure pattern			0.824
Locoregional progression	22 (62.9%)	16 (17.9%)	
LMS	10 (28.6%)	10 (34.5%)	
Locoregional progression and LMS	3 (8.6%)	3 (10.3%)	
LMS before or after bevacizumab therapy	14 (40.0%)	20 (69.0%)	0.021

Chi-square test, Mann–Whitney U test, or independent *t*-test. *p < 0.05, **p < 0.01.

GBM, Glioblastoma multiforme; KPS, Karnofsky Performance Scale; EOR, the extent of the resection; GTR, gross total resection; STR, subtotal resection; LMS, leptomeningeal spread; V50, (50 Gy irradiated brain volume, %); V60, (60 Gy irradiated brain volume, %); V80, (80 Gy irradiated brain volume, %); ORR, objective response rate.

**Figure 1:**
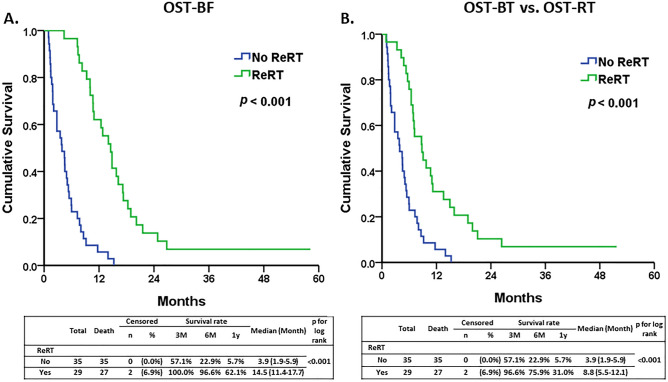
The analysis of overall survival time after bevacizumab failure (OST-BF) and a comparison of OST-BF and overall survival time after re-irradiation (OST-RT) between the two groups. (**A**) The Kaplan–Meier curves for OST-BF in none-ReRT and the ReRT group. The ReRT group exhibited significantly longer OST-BF than the none-ReRT group (median 13.5 vs. 3.9 months, *p* < 0.001). (**B**) The ReRT group exhibited significantly longer OST-RT than the OST-BT in none-ReRT group (median 8.8 vs. 3.9 months, *p* < 0.001).

The Kaplan–Meier survival analysis revealed that the ReRT group had a significantly higher survival rate and longer median survival time than the none-ReRT group. The median overall survival time after bevacizumab failure (OST-BF) and after re-irradiation (OST-RT) was 14.5 months and 8.8 months, respectively, for the ReRT group. In comparison, the OST-BF for the none-ReRT group was 3.9 months (95% CI: 1.9–5.9, *p* < 0.001) (Fig. 1). The survival curve for the ReRT group remained higher than that of the none-ReRT group throughout the follow-up period, indicating a sustained survival benefit.

**Figure 2:**
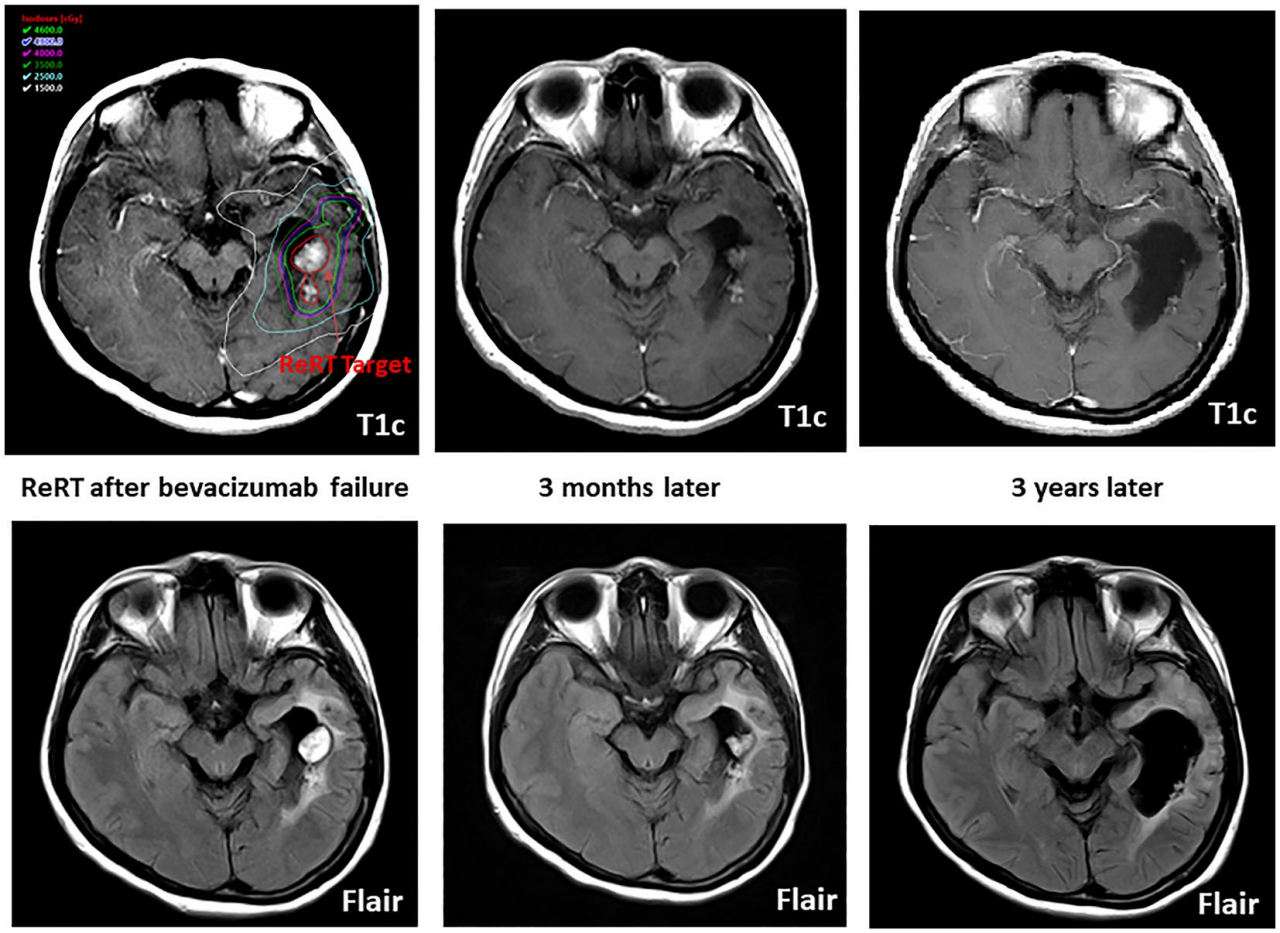
The images illustrate a long-term survivor with a high KPS in the ReRT group who had a small re-irradiation target and did not experience exacerbated cerebral edema after re-irradiation. Notably, there was no significant brain edema observed at the 3-year follow-up.

Cox univariate analysis for OST-BF in all patients identified KPS at bevacizumab failure, ORR, bevacizumab failure pattern, re-irradiation, and re-irradiation target volume as significant predictors of survival time after bevacizumab failure (Table 2). Further multivariable analysis showed that patients with a higher KPS score, good bevacizumab responder, and re-irradiation had longer survival times. Similarly, univariate analysis for OST-RT in the ReRT group revealed KPS at bevacizumab failure, bevacizumab ORR, re-surgery after bevacizumab failure, worse bevacizumab failure pattern, and re-irradiation target volume as significant predictors (Table 3). The multivariable analysis found only the re-irradiation target volume to be significant. In addition, the area under the curve (AUC) analysis showed that the re-irradiation target volume had an excellent discriminatory ability, with an optimal cutoff point of greater than 27.58 ml, a sensitivity of 74.07%, and a specificity of 100.00% (Supplementary 2). These results indicate that the re-irradiation target volume and survival time after re-irradiation may be applicable prognostic factors for these patients. Figure 2 depicts a long-time survivor with a high KPS in the ReRT group who had a small re-irradiation target and did not experience exacerbated cerebral edema after re-irradiation.

**Table 2:** Cox regression analysis of the factors for the survival time after bevacizumab failure (N = 64).

	Univariate			Multivariable		
	HR	95% CI	*p* value	HR	95% CI	*p* value
Gender						
Female	Reference					
Male	0.84	(0.50–1.42)	0.523			
Age, years	1.01	(0.99–1.03)	0.477			
KPS at bevacizumab failure	0.91	(0.87–0.96)	< 0.001**	0.93	(0.88–0.97)	0.002**
Bevacizumab ORR						
Complete response	Reference			Reference		
Partial response	2.73	(1.36–5.47)	0.005	2.26	(1.09–4.69)	0.029*
Stable	1.11	(0.26–4.78)	0.887	2.22	(0.48–10.16)	0.305
Progression	4.85	(2.48–9.48)	< 0.001**	3.41	(1.62–7.16)	0.001**
1st Surgery EOR						
GTR	Reference					
STR-PR	1.53	(0.91–2.56)	0.105			
Re-surgery after bevacizumab failure	1.27	(0.70–2.31)	0.427			
Bevacizumab failure pattern						
Locoregional	Reference			Reference		
LMS	1.12	(0.64–1.96)	0.691	1.21	(0.64–2.29)	0.560
Locoregioal + LMS	2.53	(1.03–6.17)	0.042*	1.93	(0.73–5.12)	0.184
Re-irradiation						
No	Reference			Reference		
Yes	0.32	(0.19–0.55)	< 0.001**	0.33	(0.17–0.62)	< 0.001**
Re-irradiation target						
Locoregional	Reference					
LMS	1.01	(0.45–2.23)	0.988			
Both	27.56	(1.69–448.06)	0.020*			
Re-irradiation dose	1.00	(1.00–1.00)	0.223			
Re-irradiation target volume	1.01	(1.00–1.02)	0.003**			
The time interval between re-irradiation and bevacizumab failure	1.04	(0.93–1.17)	0.513			

Cox proportional hazard regression. *p < 0.05, **p < 0.01.

HR, Hazard ratio; ORR, objective response rate; KPS, Karnofsky Performance Scale; EOR, the extent of the resection; GTR, gross total resection; STR, subtotal resection; LR, locoregional recurrence; LMS, leptomeningeal spread.

**Table 3:** Cox regression analysis of the factors for the survival time after re-irradiation (N = 29).

	Univariate			Multivariable		
	HR	95%CI	*p* value	HR	95%CI	*p* value
Gender						
Female	Reference					
Male	0.76	(0.33–1.76)	0.522			
Age, years	1.01	(0.98–1.03)	0.638			
KPS at bevacizumab failure	0.90	(0.83–0.98)	0.012*	0.94	(0.84–1.06)	0.298
Bevacizumab ORR						
Complete response	Reference			Reference		
Partial response	1.40	(0.39–4.96)	0.604	2.06	(0.39–10.85)	0.396
Stable	0.61	(0.08–4.74)	0.641	1.15	(0.13–10.05)	0.897
Progression	3.30	(1.10–9.88)	0.033*	3.76	(0.87–16.27)	0.076
1st Surgery EOR						
GTR	Reference					
STR-PR	1.30	(0.57–2.95)	0.532			
Re-surgery after bevacizumab failure	3.62	(1.43–9.17)	0.007**	2.04	(0.57–7.24)	0.271
Bevacizumab failure pattern						
Locoregional	Reference			Reference		
LMS	1.16	(0.49–2.75)	0.735	1.48	(0.15–14.40)	0.735
Locoregional + LMS	4.68	(1.20–18.23)	0.026*	8.85	(0.36–215.54)	0.181
LMS before or after bevacizumab therapy	1.50	(0.65–3.49)	0.343			
The interval between 1st surgery to re-irradiation	0.908	(0.950–1.011)	0.209			
Re-irradiation target						
Locoreional	Reference			Reference		
LMS	1.15	(0.50–2.60)	0.747	0.52	(0.05–5.60)	0.592
Both	27.87	(1.71–453.99)	0.019**	1.26	(0.02–71.62)	0.910
Re-irradiation dose	1.00	(1.00–1.00)	0.234			
Re-irradiation target volume	1.01	(1.01–1.02)	0.001**	1.01	(1.00–1.03)	0.006**

Cox proportional hazard regression. *p < 0.05, **p < 0.01.

HR, Hazard ratio; ORR, objective response rate; KPS, Karnofsky Performance Scale; EOR, the extent of resection; GTR, gross total resection; STR, subtotal resection; LR, locoregional recurrence; LMS, leptomeningeal spread.

## Discussion

Recurrent GBM presents a significant challenge for clinicians due to the limited effectiveness of current therapeutic options, such as lomustine and bevacizumab. In a phase III trial (EORTC 26101), the combination of bevacizumab and lomustine did not significantly improve overall survival compared to lomustine monotherapy, though progression-free survival did improve^4^. However, the combination therapy was associated with increased rates of adverse events and thrombocytopenia, which is a significant limitation of lomustine chemotherapy^19^. Other agents, including fotemustine, irinotecan, and regorafenib, have been investigated but lack high-certainty evidence of superiority over lomustine^20^. With its relatively minor adverse events, bevacizumab monotherapy is considered a salvage treatment to enhance the quality of life with limited survival benefits^21^. In some countries where lomustine is unavailable, bevacizumab monotherapy represents the only treatment option for recurrent GBM. The combination of re-irradiation and bevacizumab has emerged as a promising therapeutic approach for recurrent GBM patients who fail bevacizumab treatment, particularly in cases where lomustine is unavailable or ineffective.

Re-irradiation as a potential treatment for recurrent GBM has been limited due to concerns about potential neurologic toxicities. Radiation-induced brain necrosis is a known complication of re-irradiation and is associated with higher radiation doses and larger volumes of irradiated brain tissue. Previous studies have evaluated the risks of radiation necrosis in patients who received repeated stereotactic radiosurgery (SRS) or SRS after whole-brain irradiation^22–26^. A meta-analysis of fractionated radiation therapy found that the incidence of brain radionecrosis was 5% and 10% at biologically effective doses (BED) of 120 Gy and 150 Gy, respectively, for a fraction size less than 2.5 Gy^27^. In a study by McKay et al., the V40 Gy (median BED 306.67) was proposed to be predictive of radiation necrosis in patients treated with a second course of stereotactic radiosurgery (SRS) after local failure^28^. In our study, re-irradiation was primarily administered at a dose of 46 Gy in 20 fractions, resulting in an accumulated BED of 187.2 Gy to the planning target volume (PTV) and with a mean 9.4% brain V80 (BED 133.3 Gy). This treatment regimen appears to present an acceptable theoretical risk for radionecrosis. Furthermore, our study identified the re-irradiation target volume as an important prognostic factor for survival time after re-irradiation, with smaller target volumes associated with better survival outcomes and, theoretically, fewer neurotoxicities. This finding is consistent with previous research suggesting that the extent of disease and target volume may affect the outcome of re-irradiation treatment in patients with recurrent GBM^29,30^.

As recurrent GBM poses a significant challenge for patients, alternative treatment options include tumor treating fields therapy (TTFields)^31^, re-surgery^32^, or investigational agents^33–35^. Continued bevacizumab therapy beyond the second progression of GBM has also been studied as an alternative treatment option^36^. In this study, the addition of re-irradiation to bevacizumab treatment in patients who had failed bevacizumab monotherapy demonstrated a survival benefit. This combination therapy could provide an additional treatment option for patients, especially in settings where lomustine is unavailable or ineffective. The synergistic effects of re-irradiation and bevacizumab may improve local tumor control and maintain anti-angiogenic properties, ultimately leading to better outcomes. Future studies should focus on optimizing the dose and fractionation schemes for re-irradiation and identifying the ideal patient population that may benefit most from this combination therapy. Additionally, the integration of novel therapies, such as immune checkpoint inhibitors or targeted molecular therapies, could be explored in combination with re-irradiation and bevacizumab to potentially enhance the efficacy of treatment for recurrent GBM.

However, it is important to acknowledge the limitations of this study. One limitation is the small sample size, which may restrict the generalizability of the findings. Furthermore, our study did not include various important molecular factors such as IDH status, autophagy-related genes, hsa-miR-196a-5p, and transcription factors like CASZ1 as predictors for glioma prognosis^37–40^. Incorporating these factors into predictive models, such as a nomogram, would provide a more comprehensive and accurate tool for personalized treatment decisions and improved prognosis assessment in recurrent GBM patients. Future research should aim to address these limitations and incorporate a broader range of molecular factors to enhance the predictive models' clinical utility.

## Supplementary Information

Supplementary Information.

## Data Availability

The datasets used and/or analysed during the current study available from the corresponding author on reasonable request.
