# Supplementary material for: Re-irradiation combined with bevacizumab for recurrent glioblastoma beyond bevacizumab failure: survival outcomes and prognostic factors
Source: Sci Rep. 2023 Jun 9;13:9442. doi: 10.1038/s41598-023-36290-2 (PMC10256803; doi:10.1038/s41598-023-36290-2)

Supplementary 1. Patient selection flowchart.


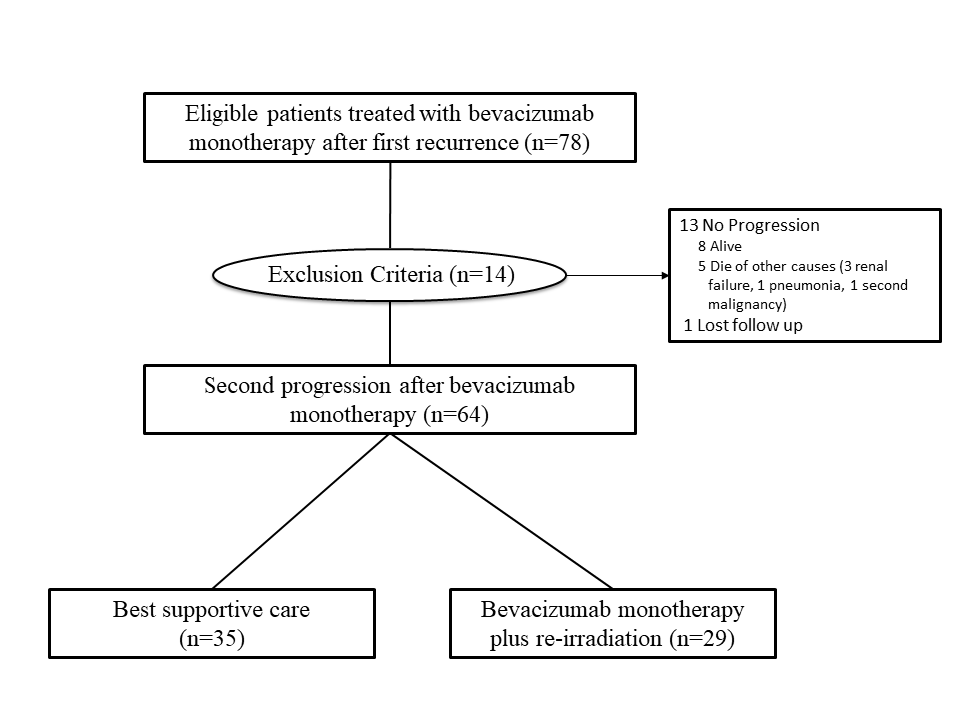


Supplementary 2. Specifically, the images demonstrate that a larger re-irradiation target volume is associated with worse treatment outcomes. This is shown by the changes in the T1c and flair MRI images, which indicate increased tumor growth and edema in the brain following treatment with a larger re-irradiation target volume.


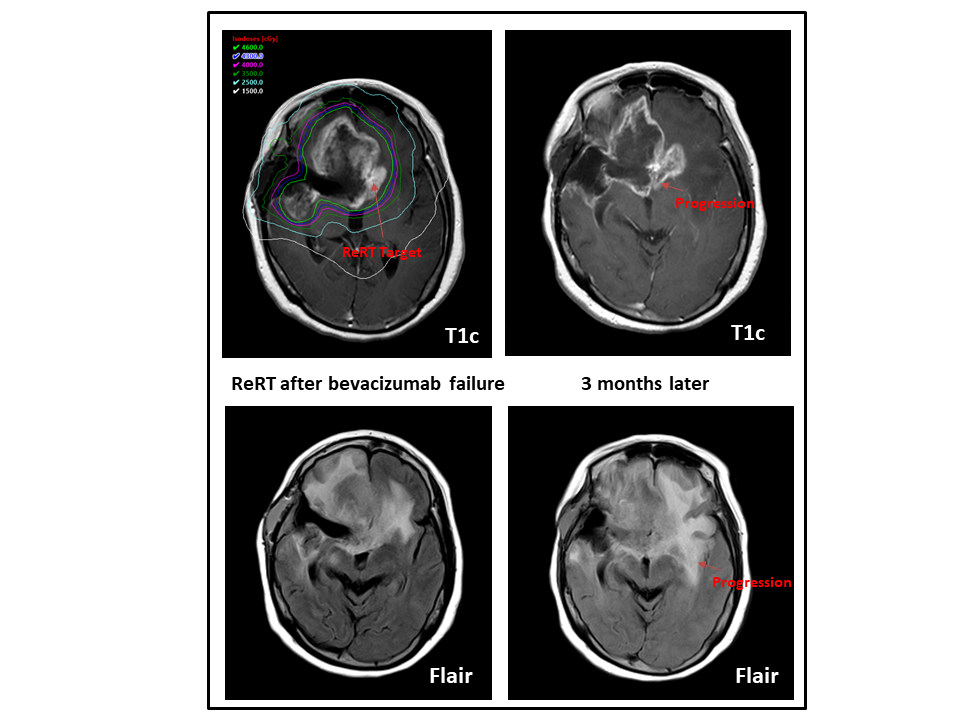


Supplementary 3. The left upper and left lower images show the T1c MRI and flair MRI, respectively, used for treatment planning. The right upper and right lower images represent the T1c MRI and flair MRI, respectively, taken 3 months after treatment. The patient in this case did not receive re-irradiation after bevacizumab failure. The smaller recurrence observed at the time of bevacizumab failure worsened after 3 months, as seen in the MRI images.


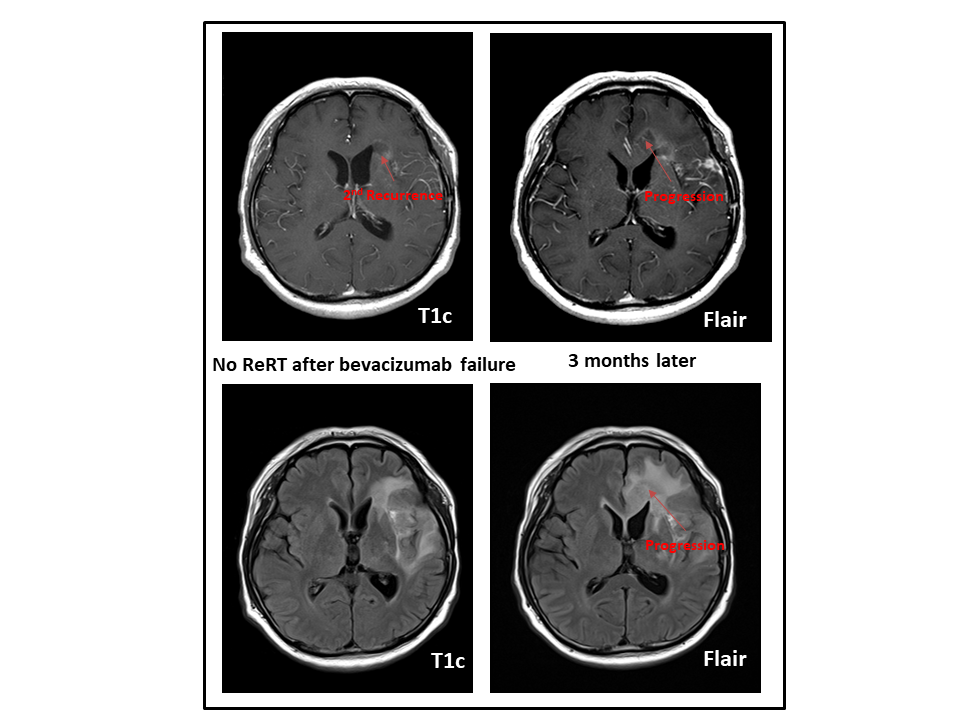

Supplement: Supplementary file 1 — Supplementary Information. [file 41598_2023_36290_MOESM1_ESM.docx]
